# Supplementary material for: The interactome of intact mitochondria by cross-linking mass spectrometry provides evidence for coexisting respiratory supercomplexes
Source: Mol Cell Proteomics. 2017 Dec 8;17(2):216–32. doi: 10.1074/mcp.RA117.000470 (PMC5795388; doi:10.1074/mcp.RA117.000470)
Supplement: Supplemental Data [file supp_RA117.000470_133922_0_supp_23978_fzffwf.zip › spectra_annotation/mito_DR_spectra_annotation/214-1-7-1-8-1.pdf]

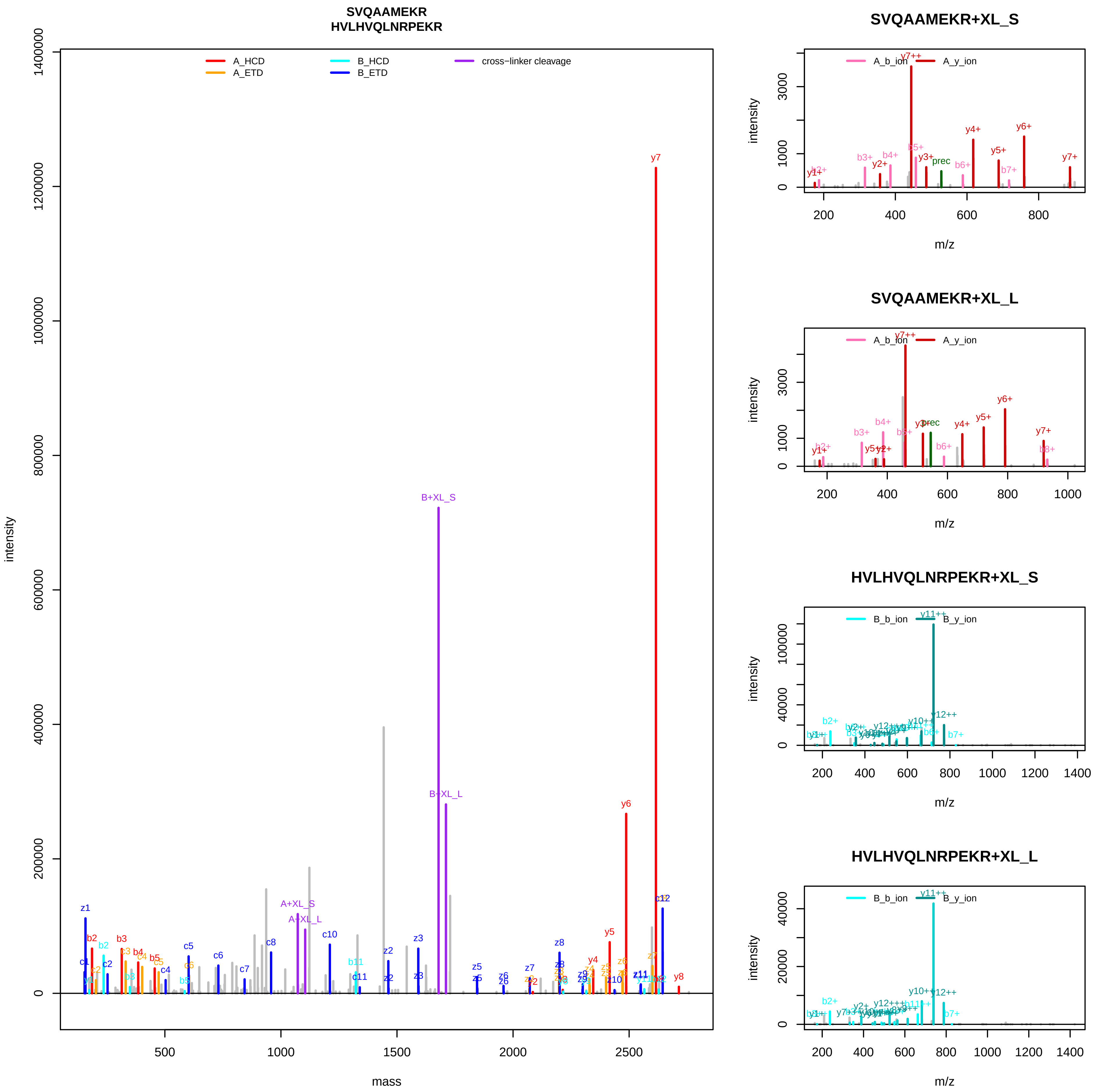

SVQAAMEKR
HVLHVQLNRPEKR
A_HCD
A_ETD
B_HCD
B_ETD
cross−linker cleavage
intensity
mass
0
200000
400000
600000
800000
1000000
1200000
1400000
500
1000
1500
2000
2500
y7
B+XL_S
B+XL_L
y6
A+XL_S
A+XL_L
c12
y5
y8
z1
b2
b3
b4
b5
c5
c6
c7
c8
c10
z2
z3
z5
z6
z7
z8
z9
z10
z11
c11
b11
y4
SVQAAMEKR+XL_S
A_b_ion
A_y_ion
m/z
y7++
y4+
y6+
y5+
y7+
b7+
b6+
prec
SVQAAMEKR+XL_L
HVLHVQLNRPEKR+XL_S
B_b_ion
B_y_ion
y11++
y12++
y10++
b2+
b7+
HVLHVQLNRPEKR+XL_L
